# Supplementary material for: Dysregulated Expression of Three Genes in Colorectal Cancer Stratifies Patients into Three Risk Groups
Source: Cancers (Basel). 2022 Aug 23;14(17):4076. doi: 10.3390/cancers14174076 (PMC9454483; doi:10.3390/cancers14174076)
Supplement: Supplementary file 1 [file cancers-14-04076-s001.zip › cancers-1836145-supplementary.pdf]

Figure S1. Classification of sCRC tumors vs non-tumoral colorectal tissues. Biplot analysis of 66 primary colorectal tumors (orange triangles) *vs.* 10 non-tumoral colorectal tissue samples (blue circles). The figure shows that, those mRNA transcripts differentially expressed in primary tumors vs. non-tumoral colorectal tissues, allowed for clear cut discrimination between both types of samples.

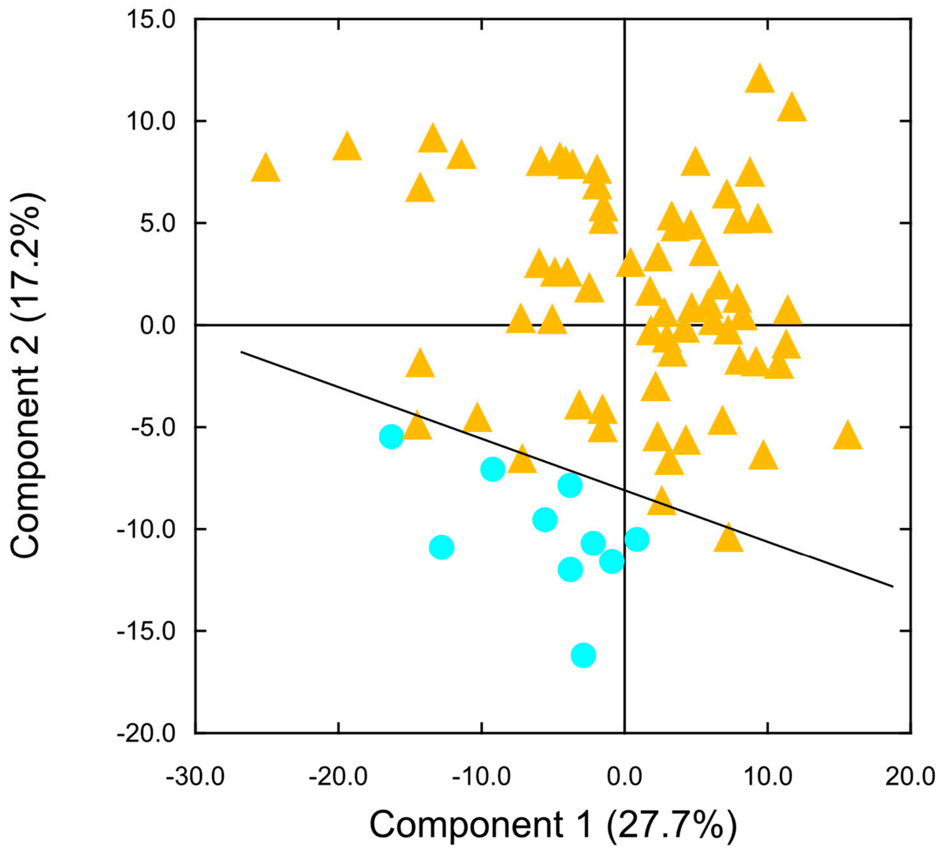

Figure S2. Validation of the impact of *BST2* and *ADH1B* (NM_001286650) expression on overall survival in an independent series of sCRC patients from the GEO database (GSE39582; n =562).

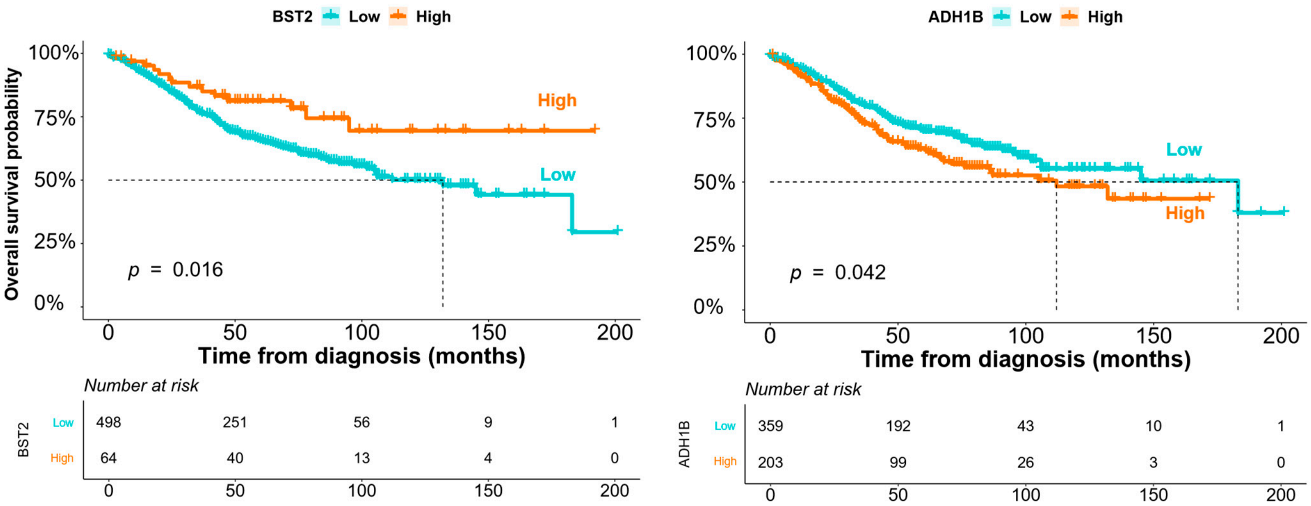

Table S1. Designed GEP panel. Primer sequencers used for each gene studied (n=28); all mRNA transcript isoforms of the genes analyzed have also been included.

Gene name	Gene ID[#]	Position	Target sequences (5'-3')
ADGRG7	NM_001308362	15-114	GAGTGGACATGGTGTTCAGGAGATTGAGGCTCAGCTAAATTCCAGCTTATTTACCTGCAGT TGCTTACAAAGTGTTTGGACATAATTGTGTAAAGCTAGG
ADGRG7	NM_032787	507-606	CCTATATGGGTTTTACTTTTGCCAGAATCCCAGTGGGCAGATATGGACCATCCTTGCAAACA TGTGGCAAGGATACTCCAAATGCGGGCAATCCAATGGC
ADH1B	NM_000668	1534-1633	CGAATCCCAGTGGAGGGGACCCTTTTACTTGCCCTGAACATACACATGCTGGGCCATTGTG ATTGAAGTCTTCTAACTCTGTCTCAGTTTTCACTGTCGA
ADH1B	NM_001286650	28-127	AAAGACTCACAGTCTGCTGGTGGGCAGAGAAGACAGAAACGACATGAGCACAGCAGGAAA AGAAAGTTGTCTGACAGAAGTTTGATGAGAGGAATTTGAC
ADH4	NM_000670	1-100	CACGGAACTCCCTGGGTAGGAGTTTGAAGCTTTCTTAACTCAGAAAGAAACTTCCAACACA GTTTCCCAAAGAAAAATGGGCACCAAGGGCAAAGTTATT
APOA1	NM_001318021	384-483	GACCTTGGCCGTGCTCTTCCTGACGGAGCCCCTGGGATCGAGTGAAGGACCTGGCCACTGT GTACGTGGATGTGCTCAAAGACAGCGGCAGAGACTATGT
APOB	NM_000384	189-288	CTGCTGGCGGGCGCCAGGGCCGAAGAGGAAATGCTGGAAAATGTCAGCCTGGTCTGTCCA AAAGATGCGACCCGATTCAAGCACCTCCGGAAGTACACAT
APOH	NM_000042	49-148	GTGCTCATCTTGTTCTCGAGTTTTCTCTGCCATGTTGCTATTGCAGGACGGACCTGTCCCAA GCCAGATGATTTACCATTTTCCACAGTGGTCCCGTTAA
BST2	NM_004335	15-114	CCTCCCCTAACTCCAGGCCAGACTCTAAAGGGGAGATCTGGATGGCATCTACTTCGTATGA CTATTGCAGAGTGCCCATGGAAGACGGGGATAAGCGCTG
CXCL14	NM_004887	242-341	TGCTGCTGCTGGCGCTGTACACCGCGCGTGTGGACGGGTCCAAATGCAAGTGCTCCCGGAA GGGACCCAAGATCCGCTACAGCGACGTGAAGAAGCTGGA
CXCL3	NM_002090	363-462	AATCATCGAAAAGATACTGAACAAGGGGAGCACCAACTGACAGGAGAGAAGTAAGAAGCT TATCAGCGTATCATTGACACTTCCTGCAGGGTGGTCCCTG
F5	NM_000130	12-111	GACAGCCCGGAGTGTGGTTAGCAGCTCGGCAAGCGCTGCCCAGGTCCTGGGGTGGTGGCA GCCAGCGGGAGCAGGAAAGGAAGCATGTTCCCAGGCTGCC
FBXO32	NM_001242463	482-581	ATTGCACCCTGGGGGAAGCTTTCAACAGACTGGACTTCTCAACTGCCATTCTGGATTCCAG AAGATTTAACTACGTGGTCCGGCCTGCCTTCAAAGGCCT

Gene name	Gene ID#	Position	Target sequences (5'-3')
FBXO32	NM_058229	290-389	TCGTGAGCGACCTCAGCAGTTACTGCAACAAGGAGGTATACAATAAGGAGAATCTTTTCAA CAGCCTGAACTATGATGTTGCAGCCAAGAAGAGAAAGAA
FBXO32	NM_148177	3-102	GTACAGGAAAGAAGCCAGTGCCCCAGGTCACCTAAGACAGGCATCAAGCTCATTCGGCAA AATCCAGTTGGGTTGTTCTAACTGGCTCATTCTTACTTGG
FER1L4	NR_119376	114-213	CACCCACGACCGACAAGTGAAGCTCACCTTTCGAGGCTTTACCCAGAAAACAAGAAAAATT CACTGTGGTCCAGAAGCAGATATCGGTGAGCTGTTCCGA
FTCD	NM_001320412	1221-1320	ACGACGATGCGGCGCCTGATCCCGCCCTTCCGCGAGGCTTCGGCCAAGCTAACCACGCTGG TGGATGCCGACGCCGAGGCCTTCACCGCCTACCTGGAAG
HPX	NM_000613	2-101	TCCTGTGGCCTCTGCAGCTCAGCATGGCTAGGGTACTGGGAGCACCCGTTGCACTGGGGTT GTGGAGCCTATGCTGGTCTCTGGCCATTGCCACCCCTCT
HRG	NM_000412	673-772	AGAGCAGATTTGTTCTATGATGTAGAAGCCTTGGACTTGGAAAGCCCGAAAAACCTTGTCA TAAACTGTGAAGTCTTCGACCCTCAGGAACATGAGAACA
IL13RA2	NM_000640	1-100	CTTCCGGATGAAGGCTATTTGAAGTCGCCATAACCTGGTCAGAAGTGTGCCTGTCGGCGGG GAGAGAGGCAATATCAAGGTTTTAAATCTCGGAGAAATG
ITIH1	NM_001166434	1-100	ATTCATCAGTGACTTTGCCGTGTGCGTGCCTGCCCAACTCCCATGCCTTCTCCCAGGGGTGC CGCTTACTAGTCCCAGCTTAAGAACAAGGATCGGAGTG
ITIH1	NM_001166435	80-179	GTGCCTACTGAGTACCAGGCCCTGTGATCAGGGCTGGGTGGGTGCTGCACACAGTAGGTGG CCAGGACACATGCTGGCTCTTGGCTGAGACTTGATTGAT
ITIH1	NM_001166436	24-123	TGAGTTGAGGGATGCAGGCATGTACACCTTCATTTGATTGATGTGGACATCTTCGAGCCCC AGGGGATCAGCAAGCTGGATGCCCAGGCCTCTTTCCTGC
ITIH1	NM_002215	8-107	GCCTTAGAGCATGGACGGTGCCATGGGGCCTCGGGGGCTGCTGTTGTGCATGTACCTGGTA TCTCTCCTCATCCTGCAGGCCATGCCTGCCCTGGGCTCG
MOCOS	NM_017947	247-346	CACTAGTGATCTCATGGAAAACACTTATGGTAATCCTCACAGCCAGAACATCAGCAGCAAG CTCACCCATGACACTGTGGAGCAGGTGCGCTACAGAATC
MYLK	NM_001321309	445-544	AAAGAAGGAGCCACCGCCAAGTTCGAAGGGCGGGAAGTTTTGCGAAGCAGCTTGGTCAGC CTGTTGTTTCCAAAACCTTAGGGGATAGATTTTCAGCTCC
MYLK	NM_053026	1526-1625	TGGAGGGCCAGAGGGATTCAGCATTCCCCAAATTTGAGAGCAAGCCCCAAAGCCAGGAGG TCAAGGAAAATCAAACTGTCAAGTTCAGATGTGAAGGGCT

Gene name	Gene ID#	Position	Target sequences (5'-3')
PCOLCE2	NM_013363	49-148	GGAGCGCGCGGGCAGCCGAGCGCCGGTGTGAGCCAGCGCTGCTGCCAGTGTGAGCGGCGG TGTGAGCGCGGTGGGTGCGGAGGGGCGTGTGTGCCGGCGC
PLG	NM_000301	633-732	ACTACTGCGACATTCTTGAGTGTGAAGAGGAATGTATGCATTGCAGTGGAGAAAACTATGA CGGCAAAATTTCCAAGACCATGTCTGGACTGGAATGCCA
PRAP1	NM_001145201	18-117	ACTCTCTACAGAGACGCGGACCCCAGACATGAGGAGGCTCCTCCTGGTCACCAGCCTGGTG GTTGTGCTGCTGTGGGAGGCAGGTGCAGTCCCAGCACCC
PRAP1	NM_145202	238-337	GTCCAGAAGCCGAAACTCTTGACCACCGAGGAGAAGCCACGAGGTCAGGGCAGGGGCCCC ATCCTTCCAGGCACCAAGGCCTGGATGGAGACCGAGGACA
RBP4	NM_001323517	12-111	GCACGAGTGCAGGGTAACTGAGCCAGGGCCGCTGGCGCATTTGGCCTGGCCGAGGCCACC CCGCGCGGCCGCTCCACTGTGCCCGAGGCTGTCCTGGAGG
RBP4	NM_001323518	11-110	AAATGAAAAACTACTTGGATGAATTATTCCAAAATTCCTGCACAAGTGGACCTCAGAAGGC AGACGGAGCGCGACTGCCGAGTGAGCAGCTTCCGAGTCA
RBP4	NM_006744	935-1034	TTTTCATTTGTGTTTATGTCTGTGCTGCAGACGGATGGGTGGGGTGCGCTTCTTTATACCAG GAGCACGTGGCTCTTTCTGACCTTTGGCCTGTTCTAGT
SALL4	NM_001318031	1179-1278	TCCAAGAAAGGGAAGGGGAAGCCACCGAACATCTCCGCGGTGGATGTCAAACCCAAAGAC GAGGCGGCCCTCTACAAGCACAAGTGTCGGAGCAGTCTCC
SERPINA1	NM_000295	1354-1453	GATGACATTAAAGAAGGGTTGAGCTGGTCCCTGCCTGCATGTGACTGTAAATCCCTCCCAT GTTTTCTCTGAGTCTCCCTTTGCCTGCTGAGGCTGTATG
SPP1	NM_000582	285-384	GGCCACATGGCTAAACCCTGACCCATCTCAGAAGCAGAATCTCCTAGCCCCACAGACCCTT CCAAGTAAGTCCAACGAAAGCCATGACCACATGGATGAT
SPP1	NM_001040060	20-119	ATGTCTGCAGCAGCATTTAAATTCTGGGAGGGCTTGGTTGTCAGCAGCAGCAGGAGGAGGC AGAGCACAGCATCGTCGGGACCAGACTCGTCTCAGGCCA
SPP1	NM_001251830	249-348	GGAAAAGCAGCACTAAAGATGTACCTACCCCTCCACAACAGATGAAACTGTGCCAGCCAA ACAACAAATGGGCATTGTCCCCAGAAGCTTGGACAAAAAG
SRPX2	NM_014467	66-165	TCTTTCTAAAACCCTTCTCTGAGAGAGGAATAACTATAGCTTCAGGGATAATATAGCTTTAA GGAAACTTTTGGCAGATGTGGACGTCGTAACATCTGGG
THBS2	NM_003247	75-174	GGAGGAGACGGCATCCAGTACAGAGGGGCTGGACTTGGACCCCTGCAGCAGCCCTGCACA GGAGAAGCGGCATATAAAGCCGCGCTGCCCGGGAGCCGCT

Gene name	Gene ID[#]	Position	Target sequences (5'-3')
*ACTB**	NM_001101	1011-1110	TGCAGAAGGAGATCACTGCCCTGGCACCCAGCACAATGAAGATCAAGATCATTGCTCCTCCTGAGCGCAAGTACTCCGTGTGGATCGGCGGCTCCATCCT
*GADPH**	NM_001256799	387-486	GAACGGGAAGCTTGTCATCAATGGAAATCCCATCACCATCTTCCAGGAGCGAGATCCCTCCAAAATCAAGTGGGGCGATGCTGGCGCTGAGTACGTCGTG
*TUBB**	NM_178014	1956-2055	TTCTAAGTATGTCCATTTCCCATCTCAGCTTCAAGGGAGGTGTCAGCAGTATTATCTCCACTTTCAATCTCCCTCCAAGCTCTACTCTGGAGGAGTCTGT

[#]NCBI reference sequence. *Control genes used
